# *Phyllanthus emblica*: a comprehensive review of its phytochemical composition and pharmacological properties

**DOI:** 10.3389/fphar.2023.1288618

**Published:** 2023-10-26

**Authors:** Arya Tjipta Prananda, Aminah Dalimunthe, Urip Harahap, Yogi Simanjuntak, Epina Peronika, Natasya Elsa Karosekali, Poppy Anjelisa Zaitun Hasibuan, Rony Abdi Syahputra, Putri Cahaya Situmorang, Fahrul Nurkolis

**Affiliations:** ^1^ Department of Surgery, Faculty of Medicine, Universitas Sumatera Utara, Medan, Indonesia; ^2^ Department of Pharmacology, Faculty of Pharmacy, Universitas Sumatera Utara, Medan, Indonesia; ^3^ Department of Biology, Faculty of Mathematics and Natural Sciences, Universitas Sumatera Utara, Medan, Indonesia; ^4^ Department of Biological Sciences, State Islamic University of Sunan Kalijaga (UIN Sunan Kalijaga), Yogyakarta, Indonesia

**Keywords:** Phyllanthus emblica, phytochemical composition, pharmacological properties, natural product, bioactive substances

## Abstract

*Phyllanthus emblica* Linn, a prominent member of the euphorbiaceae family, exhibits extensive distribution across a multitude of tropical and subtropical nations. Referred to as “Balakka” in Indonesia, this plant assumes various names across regions, such as “kimalaka,” “balakka,” “metengo,” “malaka,” and “kemloko” in North Sumatra, Ternate, Sundanese, and Java respectively. *Phyllanthus emblica* thrives in tropical locales like Indonesia, Malaysia, and Thailand, while also making its presence felt in subtropical regions like India, China, Uzbekistan, and Sri Lanka. The fruits of Balakka are enriched with bioactive constituents recognized for their wide-ranging benefits, including antioxidant, anti-aging, anti-cholesterol, anti-diabetic, immunomodulatory, antipyretic, analgesic, anti-inflammatory, chemoprotective, hepatoprotective, cardioprotective, antimutagenic, and antimicrobial properties. Comprising a spectrum of phenolic compounds (such as tannins, phenolic acids, and flavonoids), alkaloids, phytosterols, terpenoids, organic acids, amino acids, and vitamins, the bioactive components of Malacca fruit offer a diverse array of health-promoting attributes. In light of these insights, this review aims to comprehensively examine the pharmacological activities associated with *P. emblica* and delve into the intricate composition of its phytochemical constituents.

## 1 Introduction

*Phyllanthus emblica* Linn, a member of the euphorbiaceae family, is extensively distributed throughout the majority of tropical and subtropical nations. Phyllanthus is a very large genus containing approximately 550–750 species and 10 to 11 subgenera. It is endemic to equatorial southeast Asia and is found in the mixed forest of tropical and subtropical regions at elevations between 150 and 1,400 m. In Indonesia *P. emblica* is called balakka, kimalaka, kemlaka, kemloko, or malaka (Summanen, 1999; Mal and Meena, 2022). Natural products have existed since the dawn of humanity, the significance of traditional systems of medicine and particular traditional medical practices is now acknowledged worldwide. To evaluate selective pharmaceuticals of herbal origin, it is now necessary to adopt an intelligent and pragmatic approach. All parts of *Phyllanthus Emblica*, including its fruits, flowers, seeds, leaves, and bark, have been extensively utilized in numerous traditional remedies. Pharmacological studies reveals that *P. emblica* have antioxidant (Chaphalkar et al., 2017; Sheoran et al., 2019), anticancer (Ngamkitidechakul et al., 2010; Mahata et al., 2013; Gaire and Subedi, 2014; Zhao et al., 2015; Chekdaengphanao et al., 2022; Naik and David, 2023), Immunomodulatory (Jantan et al., 2019), cytoprotective (Zhang et al., 2016), anti-viral (Lv et al., 2014; Lv et al., 2015), anti-jaundice, anti-dyslipidemic (Quranayati et al., 2023), anti-aging (Wu et al., 2022), anti-apoptotic (Chekdaengphanao et al., 2022), anti-inflammatory (Wang et al., 2019), hepatoprotective (Pramyothin et al., 2006), nephroprotective (Huang et al., 2023), and anti-diabetic (Naik and David, 2023). *Phyllanthus emblica* has various constituents have been used in the formulation of numerous herbal and patent medicines (Dinesh et al., 2017). The majority of fixed oils, essential oils, and phosphatides are found in fruit seeds. Fruit, leaves, and bark are rich in tannins; bark also contains leucodelphinidin, while roots are abundant in lupeol and ellagic acid (Hussain et al., 2021). The yellowish-brown seeds of *P. emblica* contain linoleic acid, stearic acid, palmitic acid, linolenic acid, myristic acid, and oleic acid while D-myoinositol, D-fructose, and D-glucose are predominantly found in the ethanol fractions of *P. emblica* (Saini et al., 2022). *Phyllanthus emblica* contains chemopreventive compounds like lupol and glochidone, which belong to the lupine-type triterpenoids (Ramasamy et al., 2012). Additionally, it harbors antioxidant properties from compounds like mallotusinin, isomallotusinin, isostrictinin, and mallonin, along with phyllembilin, cinnamic acid, and chebulagic acid (Ahmad et al., 2021). The vitamin C content in *P. emblica* far surpasses that of common citrus fruits like lemons, oranges, and tangerines (Bajgai et al., 2006). Besides vitamin C, this fruit also contains other essential vitamins including carotene, niacin, riboflavin, and thiamine (Ghosal, 1996). For every 100 g of *P. emblica* fruit, there is an impressive vitamin C quantity ranging from 600 to 1,300 mg. Among the amino acids present, the prominent ones are glutamic acid (29.6%), proline (14.6%), aspartate (8.1%), alanine (5.4%), and lysine (5.3%) (Saini et al., 2022). *Phyllanthus emblica* fruit is rich in vitamin C (70%–72%) and includes a variety of components such as tannins, phembembaic acid (6.3%), gallic acid (5%), lipids (6%), emblicol, flavonoids, and mymic acid. The leaves of *P. emblica* contain gallic acid, chebulic acid, ellagic acid, kaempferol, kaempferol-3-o-glucoside, gallo tannin, and rutin, phosphoric acid, essential oils, linoleic acid, oleic acid, stearic acid, palmitic acid, and mystic acid. The bark of the plant contains proanthocyanidins, tannins, and leucodelphinidin. Moving on to the roots, they contain ellagic acid and lupeol (Saini et al., 2022). This thorough review focuses on the phytochemical makeup and the effects of *P. emblica*. Through an extensive exploration, this study aims to shed light on the numerous health advantages of *P. emblica*, thereby stimulating more research and progress in utilizing this herbal remedy to enhance human health.

## 2 Botanical description and taxonomy

**FIGURE 1:**
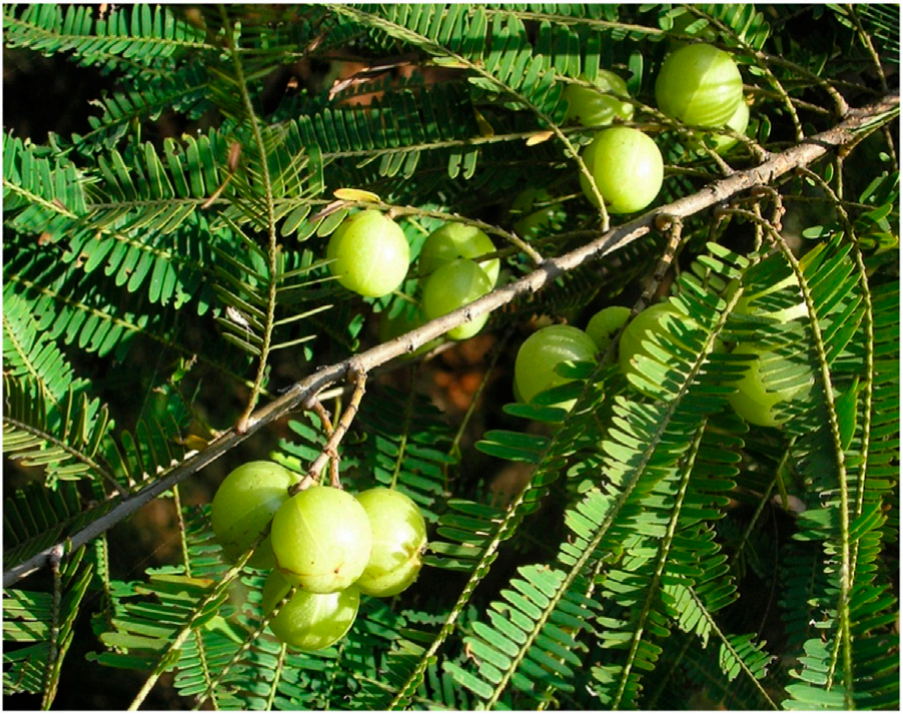
*Phyllanthus emblica*.

Balakka is a type of plant that lives in a forest on the savanna. The forest has medium-sized trees with lots of branches, and they’re about 10–20 m tall. The fruit of the balakka plant is round and has ridges. It is divided into six parts, and each part has a stone in it. The stones are about 1.8–2.5 cm across. The fruit is small and round with a tough covering. Inside, there are six seeds. The fruit looks nice–it is round and yellow. It tastes sour and astringent, which means it makes your mouth pucker a little. The balakka plant grows slowly and can climb things. It is mostly white in color. It is found in certain parts of Sumatra, which is an island in Indonesia. It likes to grow in places that are not very wet, like yards and along roads. It can also grow in places where people plant things, like farms. The balakka plant likes specific types of soil that have a pH between 6.5 and 7. There are different kinds of soil where balakka plants grow. Some soils have a gray or yellowish top layer and a red or yellow lower layer. These soils do not have a lot of nutrients, and they’re a bit acidic. The balakka plant also grows in places where the soil has lots of clay. The land where balakka grows comes from rocks and other materials in the ground. It is usually not very high above sea level, maybe around 50–350 m. The balakka plant likes places where it rains between 2,500 and 3,500 mm every year (Gantait et al., 2021). The *P. emblica* Figure 1 can be seen below.

## 3 Botanical description and taxonomy

**FIGURE 2:**
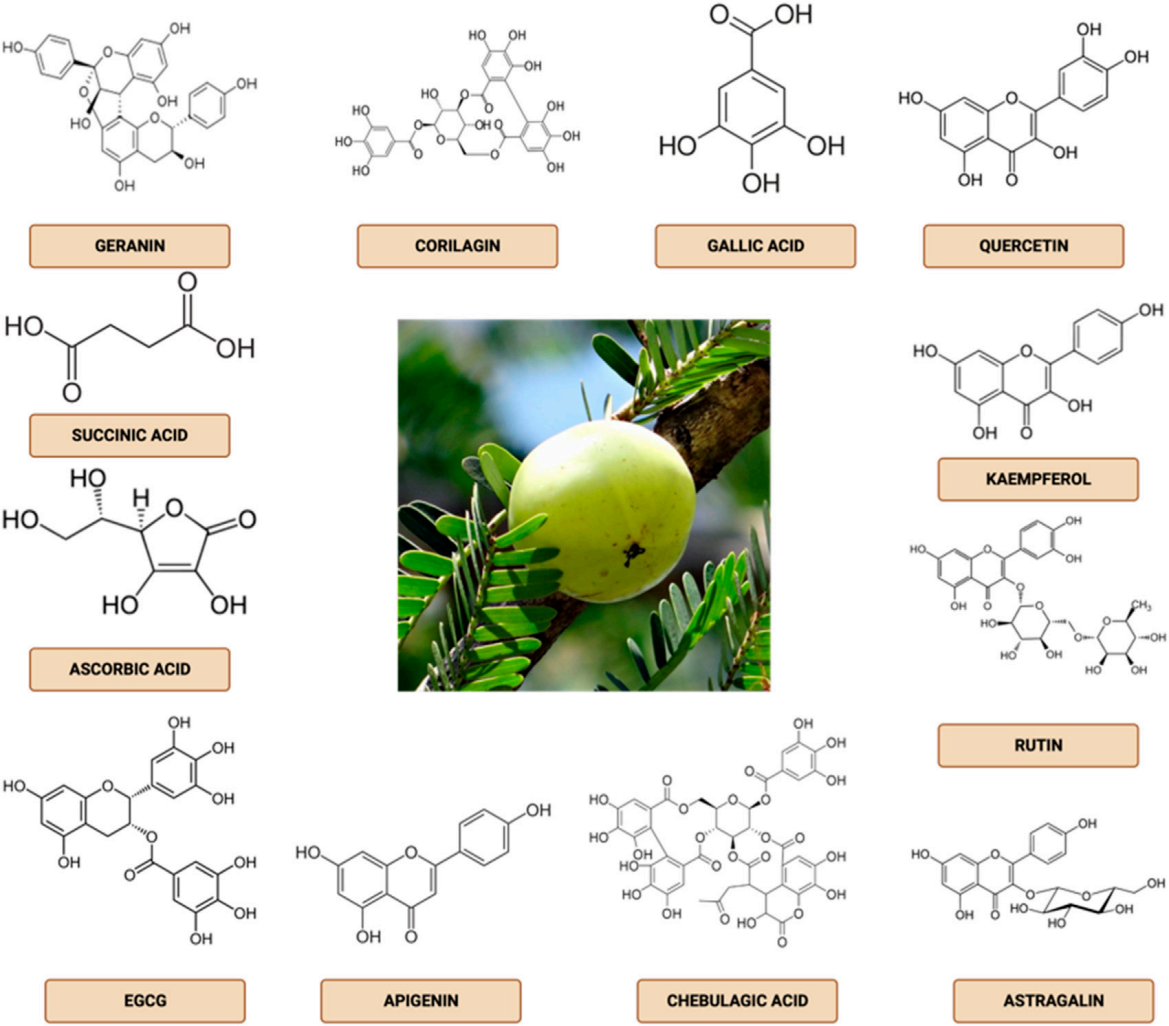
Main phytochemical components in *Phyllanthus emblica*.

The bioactive components of natural ingredients are defined as secondary metabolites with human and animal pharmacological effects. Bioactive components of *Phyllanthus emblica* fruit include a group of phenolic compounds (tannins, phenolic acids, and flavonoids), alkaloids, phytosterols, terpenoids, organic acids, amino acids, and vitamins (Yang and Liu, 2014). Emblicanin A, B, punigluconin, pedunculagin, geranin, isochorylagin, corylagin, chebulanic acid, chebulagate acid, isostrictinin, gallic acid, mucous acid lactone gallate, digalloylglucose, methyl gallate, ethyl error, monogalloyl glucose, putanjivin A, galloil-HHDP-glucose, and elaeocarpusin are hydrolyzed tannins found in *P. emblica* fruit (Usharani et al., 2013). Other bioactive substances include gallic acid, ellagic acid, chlorogenic acid, malic acid, chebulic acid, and cinnamic acid. Additionally, *P. emblica* fruit contains flavonoid compounds like quercetin, kaempferol, and routine (Jagdale et al., 2021). This plant contains phytochemicals such as fixed oils, phosphatides, essential oils, tannins, minerals, vitamins, aminoacids, fatty acids, and glycosides, among others (Chanda et al., 2020). *Phyllanthus emblica* has been found to contain linolenic, linoleic, oleic, stearic, palmitic, and myristic acids. Sugar residues consist of D-glucose, D-fructose, D-myo-inositol, D-galacturonic acid, D-arabinosyI, D-rhamnosyl, D-xylosyI, D-glucosyI, D-mannosyl, and D-galactosyI (Ahmad et al., 2021). The results of our previous study indicate the presence of alkaloids (specifically phyllantidine and phyllantine) and tannins (including chebulagic acid, chebulinic acid, punigluconin, emblicanin A, ellagic acid, emblicanin B, 1-O-galloyl-b-D-glucose, ellagic acid, ellagotannin, 3-ethylgallic acid, corilagin, pedunculagin, trigallayl glucose, 3,6-di-O-galloyl-D-glucose, and 1,6-di-O-galloyl-b-D-glucose) as determined by chromatographic and infra-red spectral analysis. Additionally, the presence of flavonoids, including kaempferol-3-O-a-L-(600-ethyl)-rhamnopyranoside, quercetin, acylated apigenin glucoside, and kaempferol-3-O-a-L-(600-methyl)-rhamnopyranoside, was also documented (Halim et al., 2022). The chemical structure of phytochemical composition in *P. emblica* can be seen in Figure 2 and Table 1 below.

**TABLE 1:** Phytochemical components of *Phyllanthus emblica*.

No.	Extraction method	Analytic	Bioactiv compund identified	Part of the plant	References
		Technique			
1	etylacetate	GC-MS	- Citronellyl Propionate	fruit	Acharya (2016)
			- 1-Methyl-4 Isopropyl-Cyclohexyl 2-Hydroperfluorobutanoate		
			- Citronellyl Acetate		
			−3,7,11,15-Tetra Methyl-2 Hexadecen-1-Ol		
			- Bicyclo (2.2.1) - Heptane,1,3,3-Trimethyl-		
			- 7-Octadecyne,2-Methyl		
			- Bicyclo(2.2.1) Heptane, 1,7,7-Trimethyl-		
			- Bicyclo(3.1.1) Heptane,2,6,6-Trimethyl-		
			- Bicyclo(2.2.1) Heptane,2,2,3-Trimethyl-Endo-		
			- Cyclohexane, 1-Methyl-4-(1-Methylethenyl)- Cis-		
2	petroleum ether	GC-MS	- Hentriacontane	Leaf	Elangovan and Irulappan (2015)
			- Dotriacontane		
			- Tetracontane		
			- Tritetracontane		
			- Pentacosane		
			- Octadecanoic Acid		
			- Methyl Ester		
			- Vitamin E		
3	n-hexane, ethyl acetate, and methanol solvents	GC-MS	- 9-Octadecene	Stem bark	Quranayati et al. (2023)
			- Methyl Palmitate		
			- 1-Octadecene		
			- 1-Tetracosanol		
			- Docosanoic Acid		
			- Cyclopropane, 1-(1-Hydroxy-1-Heptyl)-2-Methylene-3-Pentyl		
			- 1-Pentadecene		
			- 1-Hexadecene		
			- 9-Eicosene, (E)-		
			- Neophytadiene		
4	methyl salicylate	GC-MS	- 2-Methyl Butyl Acetate	fruit	Amir, et al. (2014)
			- Isopropyl,2-Methyl Butyrate		
			−2,4-Hexadienol		
			- Benzaldehyde		
			- Menthane		
			- Decane		
			- Butyl Cyclohexane		
			- Butyl Cyclohexene		
			- Acetophenone		
			- Undecane		
5	N-Hexane, Ethyl Acetate, and Ethanol	GC-MS	- 2-Furanmethanol	Leaf	Asmilia et al. (2020)
			- 1h-Cyclopropaanaphthalene		
			- Trans-Caryophyllene		
			- Cyclohexane		
			- Caryophyllene		
			- Sativen		
			- Delta-Guaiene		
			- Tetradecanoic Acid		
			- Octadecanal		
			- 3-Eicosyne		
6	Methanol	GC-MS	- Octyl-Β-D-Glucopyranoside	fruit	Dinesh et al. (2016)
7	Ethanol	GC-MS	- Galacturonic Acid	fruit	Li et al. (2019)
			- Glucuronic Acid		
			- The Monosaccharide Standard		
			- Hydroxylamine Hydrochloride N-Propylamine		
			- 3-Phenylphenol		
			- Trifluoroacetic Acid		
8	Ethanol and methanol	GC-MS	−1,2,3-Benzenetriol	fruit	Nair et al. (2020)
			- Hexadecanoic Acid		
			- 2-Tert-Butyl-4-Iso Propyl-5 Methyl Phenol		
			- 9-Octadecenoic Acid		
			- Octadecanoic Acid		
9	Methanol	GC-MS	−1,5-Hexanediol	leaf	Abdel-Hady et al. (2022)
			- D-Mannose		
			−1,3-Dimethylindole		
			−24,25-Dihydroxy Vitamin D		
			- Pyrrolizin-1,7-Dione-6- Carboxylic Acid, Methyl (Ester)		
			- Phenol,2,4-Di-Tert-Butyl-		
			- Menthol,1'-[Butyln-3-One-1- Yl)-, (1r, 2s, 5r)		
			−10-Heptadecen-8-Ynoic Acid, Methyl Ester. (E)		
			- Vitamin A Palmitate		
			- Cis-11-Eicosenoic Acid		
10	Methanol	GC-MS	−2,4-Dimethylfuran	fruit	Akter et al. (2022)
			- 4-(2-Hydroxyethyl)-3-Methyl-2-Pyrazolin-5-One		
			- Trans-2,3-Epoxyoctane		
			- 2-Furancarboxylic Acid, 2-Ethylhexyl Ester		
			- Heptanoic Acid, 3-Hydroxy-, Methyl Ester		
			- 2-(3-Methylguanidino)Ethanol D-Glycero-D-Ido-Heptose		
			- Paromomycin		
			- Octadecanoic Acid		
			−9,9-Dimethoxybicyclo[3.3.1 ]Nona-2,4-Dione		
			- N-Propyl Nonyl Ether		
11	Ethanol, acetone, n-hexane	GC-MS	- Succinic Acid Dimethyl Ester	fruit	Harahap et al. (2019)
			- Dimethyl Pentanedioate		
			- Dimethyl Adipate		
			- Methyl Adipate		
			−1,3-Dioxolane-4-Methanol		
12	Ethanol	HPLC	- Quinic Acid	fruit, bark, leaf	Kumar et al. (2017a)
			- Caffeic Acid		
			- Gallic Acid		
			- Vanillic Acid		
			- Gentisic Acid-O-Hexoside -		
			- (+)-Catechin		
			- Brevifolincarboxylic Acid		
			- Epicatechin		
			- Ellagic Acid-O-Dihexoside		
			- Ellagic Acid-O-Hexoside		
13	Ethanol	HPLC	- Mucic Acid- 1,4-Lactone- 3-0-Gallate	fruit	Li et al. (2019)
			- Hamamelitannin		
			- Isocorilagin		
			- Ethyl Gallate		
			- Methyl Gallate		
			- Ellagic Acid		
			- Quercetin-3-O-Rhamnoside		
			- Undefined		
14	methanol	HPLC	- B-Glucogallin	fruit	Kumar et al. (2015)
			- Trigalloylglucose		
			- Geraniin		
			- Gallic Acida		
			- Castalin		
			- Trigalloylglucose (Isomer)		
			- Corilagin		
			- Protocatechuic Acida		
			- Methylgallate		
			- P-Coumaric Acid		
15	methanol	HPLC	- Digallic Acid	fruit	Balusamy et al. (2019)
16	Ethanol	HPLC	- Gallic Acid	fruit	Li et al. (2020)
			- Fisetin		
17	Ethanol	LC/MS	- 5-Hydroxyisophthalic Acid	fruit	Wu et al. (2022a)
			- Amlaic Acid		
			−3,5-Dihydroxybenzoic Acid		
			−10-Gingerdione		
			- 6-Methylgingediacetate		
			- Ethyl Gallate		
			- (-)-1,10-Epoxy-Guaia-11-Ene		
			- 7alpha-Hydroxycholesterol		
			- Irisoquin F		
			−2′-Hydroxycinnamaldehyde		
18	Ethanol	HPLC	- Gallic	fruit	Kumnerdkhonkaen et al. (2018)
			- Phydroxybenzoic		
			- Vanillic		
			- Syringic		
			- P-Coumaric		
			- Ferulic		
			- Sinapinic Acids		
19	Methanol	HPLC	- L-Ornithine	fruit	Luo et al. (2022)
			- Taurine		
			- Glucose 1-Phosphate		
			- Deoxycytidine		
			- Methylmalonic Acid		
			- 3-Hydroxy-3- Methylbutanoic Acid		
			- 5-Hydroxytryptamine		
			- N-Lactoyl-Phenylalanine		
			- 2-(3,4-Dimethoxyphenyl) Ethanamine		
			- Indoleacrylic Acid		
20	Methanol	LC/MS	- L-Ornithine	leaf	Kiran et al. (2021)
			- Taurine		
			- Glucose 1-Phosphate		
			- Deoxycytidine		
			- Methylmalonic Acid		
			- 3-Hydroxy-3- Methylbutanoic Acid		
			- 5-Hydroxytryptamine		
			- N-Lactoyl-Phenylalanine		
			- 2-(3,4-Dimethoxyphenyl) Ethanamine		
			- Indoleacrylic Acid		
21	Ethanol	HPLC	- Gallic Acid	fruit	Gong et al. (2020)
			- Methyl Gallate		
22	Ethanol and Methanol	LC/MS	- Arbamoyl Phosphate	fruit	Kumar et al. (2017b)
			- L-Methionine; Methionine; L-2-Amino-4methylthiobutyric Acid		
			- Pyridoxamine Phosphate		
			- Sulfate Derivative Of Norepinephrine		
			- Nicotinate D-Ribonucleoside		
			- 4-Hydroxy-All-Trans-Retinyl Acetate		
			−5(S),6(S)-Epoxy-15(S)-Hydroxy-7e,9e,112,13e- Eicosatetraenoic Acid		
			- Prostaglandin B1		
			- N6-D-Biotinyl-L-Lysine; Biocytin; Epsilon-N-Biotinyl- L-Lysine		
			−12-Oxo-20-Dihydroxy-Leukotriene B4		
23	Ethanol	GC-MS	- Fatty Acids And Fatty Acyl Esters	fruit	Khaled et al. (2018)
			- Fatty Acyl Esters		
			- Phthalides		
			- Terpenes		
			- Sterol		
			- Palmitic Acid And Palmitic Acid Methyl Ester		
			- Linoleic Acid		
			- Stearic Acid		
			- Elaidic Acid		
24	Ethanol	HPLC	- Hypophyllanthin	fruit, leaf	Kumar et al. (2017c)
			- Phyllanthin		
			- Gallic Acid		
			- Ellagic Acid		
			- Quinic Acid		
			- Chebulinic Acid		
25	Methanol	HPLC	- Mucic Acid	fruit	Yang et al. (2012)
			- Mucic Acid Lactone		
			- Malic Acid		
			- Mucic Acid Gallate		
			- Chebulic Acid		
			- Mucic Acid Digallate		
			- Mucic Acid Lactone Gallate		
			- Galloylglucose		
			- Mucic Acid Methyl Ester Gallate		
			- Gallic Acid		
26	Ethanol	HPLC	- Quercetine	fruit	Halim et al. (2022)
			- Betaine		
			- Trigonelline		
			- Stearamide		
			- Ellagic Acid		
			- Myricitrin		
			- Myricetin		
			- Leucine		
			- Kaempferol		
			- Α-Linoleic Acid		
27	Methanol	HPLC, LC/MS	- Ellagic Acid	fruit	Muthusamy et al. (2017)
			- Phyllanthin		
			- Hypophyllanthin		
			- Niranthin		

## 4 Traditional medicine use

Balakka is a component that is frequently used in traditional medicine. This plant has been used in India to treat cancer, diabetes, liver, cardiac problems, and anemia. The fruit of the Balakka tree contains chromium, zinc, and copper. Chromium exhibits substantial antidiabetic activity in a variety of experimental diabetes models. Additionally, chromium compounds can enhance the fat metabolism of diabetic rats. The fruit of balakka is used as a treatment for tuberculosis and as an anti-aging agent. The fruit of balakka contains tannin, which has antibacterial properties, and vitamin C, which has antioxidant properties. In addition, it has been demonstrated and investigated that balakka is one of the anti-cancer plants. Balakka plant flavonoids and phenols have antioxidant properties because they can capture free radicals (Vauzour et al., 2008). One of the benefits of antioxidants is their ability to prevent degenerative diseases such as diabetes mellitus caused by oxidative stress caused by the deterioration of organ cells or body systems. Besides having medicinal properties, the natural benefits of balakka include its fruit for cars, candy, jelly jam, leather contains colorants that can run out as a blue dye in various fabrics only, tanneries, furniture and agricultural implements, and firewood. So balakka can be used for a wide variety of applications healthcare or herbal medicine, food and beverage, cosmetic, industry, dyeing, tanning, etc. According to the Wealth of India, *Phyllanthus emblica* seeds have been documented as a potential remedy for asthma, bronchitis. The juice that is released during the harvesting of fruit is also utilized as an ocular rinse and for the management of ocular inflammation. According to Chopra *P. emblica* has been recognized for its notable wound healing capabilities, making it a potential treatment option for snakebites and scorpion stings. The fixed oil included in FPE has been utilized in traditional formulations as a hair tonic to enhance hair growth and pigmentation (Chopra, 1958; Bruno, 1984).

## 5 Pharmacological activity of balakka (*Phyllanthus emblica*)

### 5.1 Antioxidant activity

**FIGURE 3:**
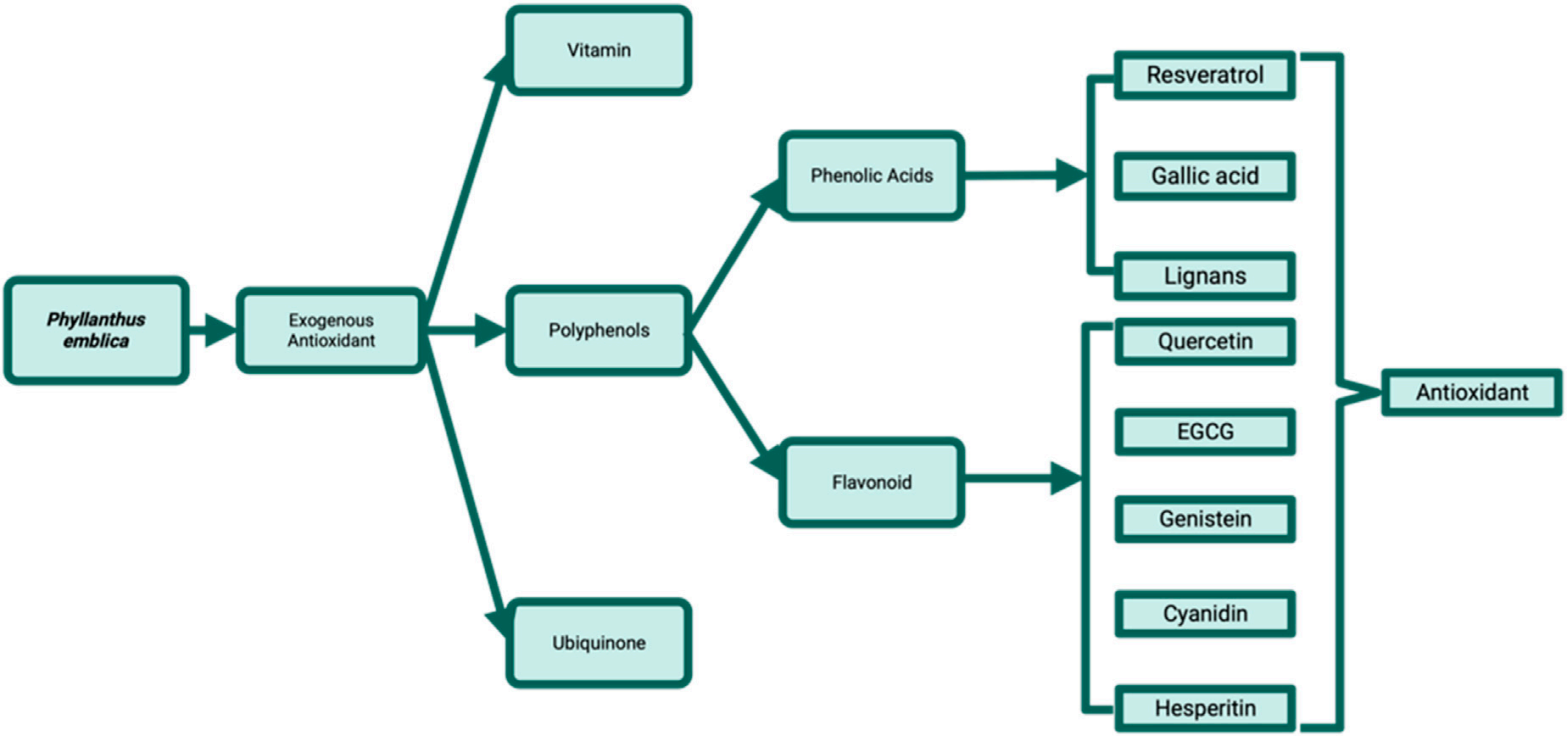
Antioxidant of Phyllanthus emblica.

Oxidative stress is associated with an increased generation of free radicals or a reduction in antioxidant content. The observed phenomenon indicates a disturbance in the equilibrium between pro-oxidant and antioxidant molecules. Pro-oxidants and free radicals are characterized by the presence of several unpaired electrons, rendering them unstable and highly reactive towards other substances. *Phyllanthus emblica* has antixoidant capacity, according to previosuly study showed that methanol extract of *P. emblica* exhibited appreciable *in vitro* antioxidant activity for scavenging the DPPH radical (IC_50_ = 39.73 ± 2.12 μg/mL), nitric dioxide (IC_50_ = 39.14 ± 2.31 μg/mL) and moderate antioxidant activity for lipid peroxidation (IC_50_ = 84,10 ± 3.04 μg/mL). *Phyllanthus emblica* also demonstrated recovery ability in CCl_4_ treated rats by elevating the level of catalase, superoxide dismutase, glutathione peroxidase and glutathione in the rats’ pulmonary. The pharmaceutical activities of *P. emblica* might be attributed to by active phyto-constituents such as gallic acid, caffeic acid, kaempferol and rutin (Tahir et al., 2016). Another study also revelaed that the IC_50_ of the *P. emblica* branches, leaves and barks aqueous extract were respectively (6.92 ± 0.22) μg/mL), (7.72 ± 0.25) μg/mL), and (6.54 ± 0.27) μg/mL). These results showed slightly lower than the IC_50_ of the ascorbic acid (8.06 ± 0.01) μg/mL) (Iamsaard et al., 2014). The antioxidant capacity of *P. emblica* were addressed by phytochemical components such as reseveratrol, gallic acid, lignans, quercetin, EGCG, genistein, cyanidin, and hesperitin (Figure 3).

### 5.2 Antiinflammatory activity

**FIGURE 4:**
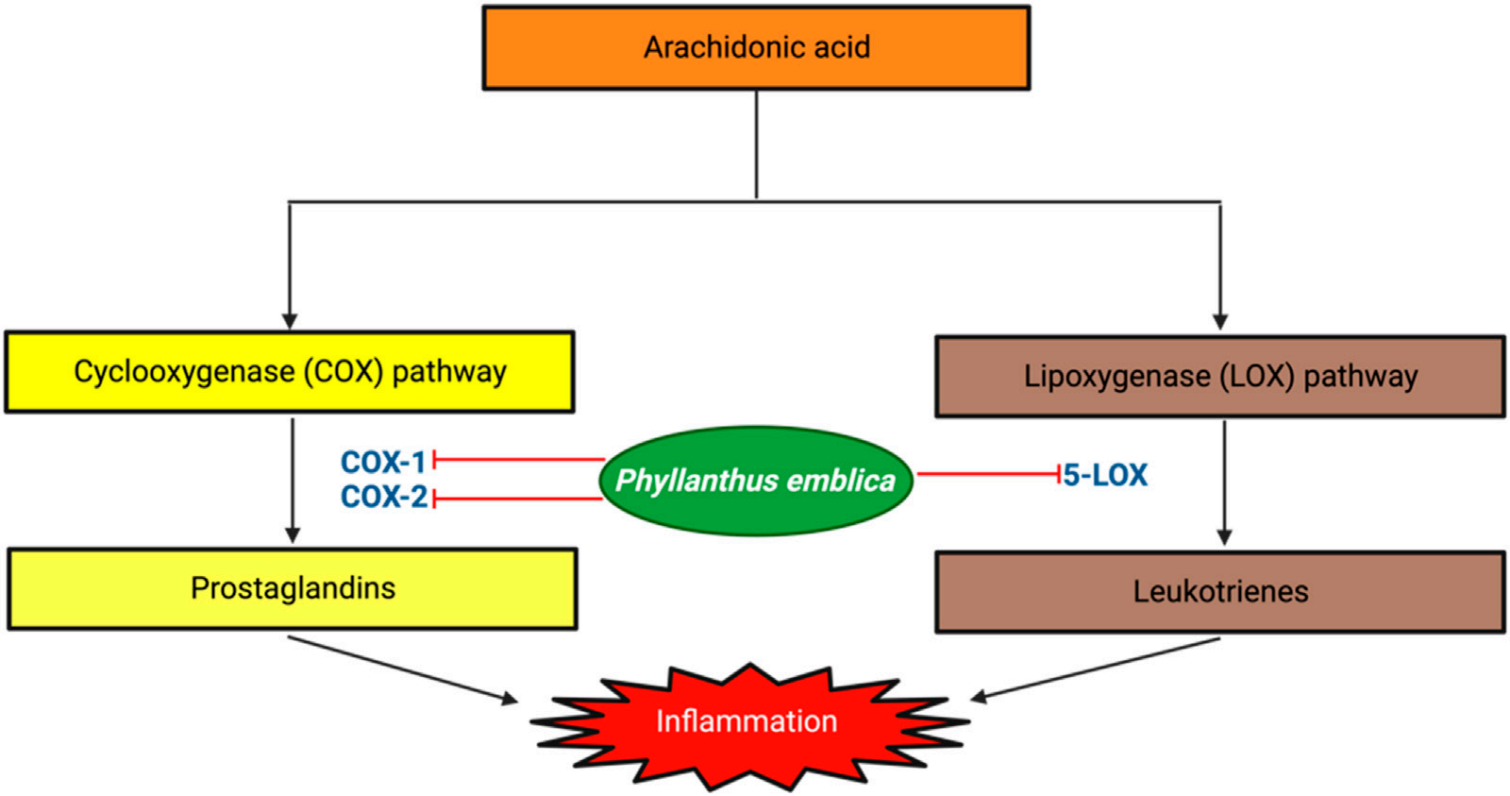
Antiinflammatory of *Phyllanthus emblica*.

Inflammation serves as a crucial innate immune response of the organism to external stimuli, such as injury or infection caused by pathogens (Jubaidi et al., 2021). Inflammation is an essential immunological response that enables the body to endure and recover from injury. Inflammation is recognized as a beneficial pathological process due to its significant involvement in restorative, healing, and aggressive mechanisms, particularly in resisting stress generated by pathogens and harmful situations (Rakha et al., 2022). The phenomenon of inflammation is intricate and encompasses a multitude of cellular responses, mostly categorized as acute and chronic inflammation. Acute inflammation serves as a protective mechanism for the body, facilitating the healing of injuries and defending against microbial invasion. In contrast, chronic inflammation specifically targets essential cells, molecules, and organs, thereby contributing to the onset and progression of diverse chronic pathologies such as cardiovascular disease, skeletal muscle disorders, inflammatory bowel disease, diabetes, cancer, and neurological diseases (Ferraz et al., 2020). Consequently, chronic inflammation also exacerbates the aging process The fruit extracts of *Phyllanthus emblica* showed a significantly hight dose-dependent inhibition of Nitric Oxide (NO) and COX-2. NO is a vital immune-signaling pathways molecule. NO overproduction could cause numerous pathological disorders and abnormalities, such as serious inflammation, cardiovascular related injury, and oxidative stress. *Phyllanthus emblica* extracts exhibited dose-dependent NO inhibition in LPS-stimulated RAW264.7. At 50 and 100 μg/mL concentration, the 95% ethanolic extract exhibited significantly higher NO inhibition (49.1%) compared to hot water and commercial extract. The extracts of *P. emblica* exhibited significantly higher COX-2 inhibition compared to hot water and commercial extract. The highest COX-2 inhibition was shown at 10 μg/mL concentration (46.4%). COX-2 inhibition may control inflammation in inflammatory diseases and abnormalities. *Phyllanthus emblica* exhibited its anti-inflammatory activities by inhibiting NO production to avoid excess NO production in macrophage cells and COX-2 enzyme (Li et al., 2022). The mechanism by which Phyllanthus emblica exerts its anti-inflammatory effects involves the inhibition of key enzymes involved in the inflammatory process, namely, COX-1, COX-2, and 5-LOX. These enzymes play pivotal roles in the synthesis of pro-inflammatory mediators, and their suppression by *P. emblica* contributes to the reduction of inflammation. This multifaceted mechanism underscores the potential therapeutic value of Phyllanthus emblica in alleviating inflammatory conditions (Figure 4).

### 5.3 Immunomodulatory activity

An immunomodulator is a constituent that possesses the ability to modulate the immune system, encompassing both innate and adaptive immunological responses (Oo et al., 2021). At now, there is a growing interest in investigating the potential of bioactive chemicals obtained from medicinal plants as agents for modulating the immune system in scientific research. Inflammation can be described as a physiological process that occurs within the human body as a result of immunological responses to combat pathogens (Zaragozá et al., 2020). The human body consists of mechanisms that serve to protect against external pathogens, such as bacteria and viruses, as well as to facilitate tissue repair and initiate the healing process following an injury. Nevertheless, an overabundance of inflammation exacerbates functional impairment, causes tissue damage, and leads to pain and discomfort (Bayat et al., 2021; Masad et al., 2021; Han et al., 2022). *Phyllanthus emblica* enhanced the effectiveness of the immunomodulatory system by raising blood levels of CD4, CD8, CD16, CD19, IgM, and IgG as well as albumin and globulin levels in the serum. In comparison to all experimental groups, the *P. emblica* group at a dose of 250 mg/kg b.wt. exhibited the most appreciable outcomes to increase immunity (Bakr and Naga, 2020). The aqueous extract of *P. emblica* fruit possessed a dose-dependent immunomodulatory activity to albino rats with a dose of 100 and 200 mg/kg for 19 days. The fruit extracts significantly increased the hemagglutination antibody titer, leukocytes count, the percentage of lymphocytes distribution, and delayed hypersensitivity in mice (Nirala et al., 2020).

### 5.4 Antidiabetic activity

**FIGURE 5:**
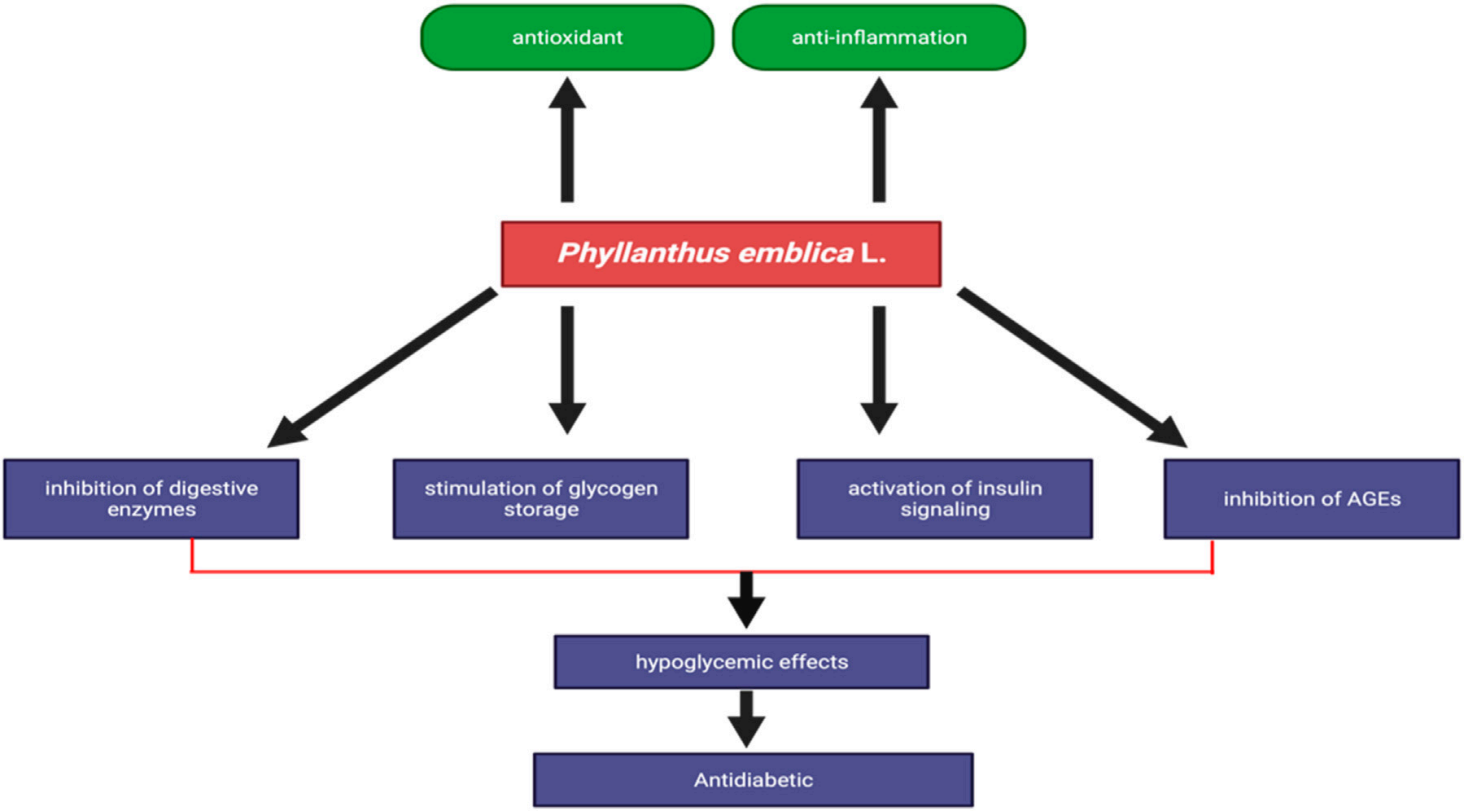
Antiinflammatory of *Phyllanthus emblica*.

Diabetes mellitus, a prevalent endocrine metabolic illness, has resulted in substantial morbidity and death as a consequence of both microvascular consequences (retinopathy, neuropathy, and nephropathy) and macrovascular complications (heart attack, stroke, and peripheral vascular disease) (Hussain et al., 2021). The human body is equipped with both enzymatic and non-enzymatic antioxidative systems that serve to mitigate the production of reactive oxygen species, which have been implicated in the development of several degenerative disorders, such as diabetes. The prevalence of the disease is experiencing a rapid escalation on a global scale, impacting various regions across the globe (Shamsudi et al., 2022). Individuals with diabetes experience elevated levels of blood glucose due to a lack of insulin. Type 2 diabetes, also known as non-insulin-dependent diabetes mellitus, is the predominant manifestation of the condition, including approximately 90%–95% of cases characterized by insufficient insulin production or utilization (Bitew et al., 2021). According to the World Health Organization, it is projected that the diabetic population would experience a substantial growth, potentially reaching 300 million or more individuals by the year 2025 (Ansari et al., 2022). Presently, the therapeutic options for diabetes encompass insulin as well as a range of oral antidiabetic medications, including sulfonylureas, biguanides, and glinides (Kalaitzoglou et al., 2019). The presence of numerous significant unfavorable effects necessitates the exploration of more efficacious and less hazardous hypoglycemic drugs, rendering it a crucial field of inquiry (Islam et al., 2023). According to Bashir et al. (2018), the ethanolic extract of *P. emblica* is a potent remedy to reduce glucose level. In diabetic rats, the glucose level significantly decreased (166 ± 0.7 mg/dL) after being treated with 80 mg/kg *P. emblica* compared to untreated diabetic rats (380 ± 0.7 mg/dL). The ethanolic extract of *P. emblica* contains tannin, an effective agent to prevent adipogenesis and increase glucose uptake by increasing insulin sensitivity towards peripheral tissues (Gul et al., 2022). *Phyllanthus emblica* has several mechanism causes hypoglycemia which are inhibition of digestive enzymes, stimulation of glycogen storage, acativation of insulin signaling, and inhibition of AGEs (Figure 5.)

### 5.5 Hepatoprotective activity

One of the most prevalent chronic liver diseases, NAFLD (Non-alcoholic fatty liver disease) is closely associated with metabolic syndrome and refers to the accumulation of hepatic steatosis that is not brought on by excessive alcohol consumption. *Phyllanthus emblica*, a rich source of gallic acid and many known medically phytochemicals and acids. *Phyllanthus emblica* fruit exhibits *in vitro* inhibitory activity on hepatic steatosis and liver fibrosis (Paik et al., 2020). The gallic acid content is also *in vivo* proven to improve high fat diet (HFD)-induced dyslipidemia, hepatosteatosis, and oxidative stress. Huang et al. initiated a research project aiming to evaluate the hepatoprotective effect of the aqueous extract of *P. emblica* L. fruit (WEPE) on NAFLD in an animal model. The findings revealed that WEPE significantly reduce body weight, peritoneal fat and epididymal fat in rats treated with HFD, as well as increase antioxidant enzyme activities and improve steatosis by increasing *adiponectin* in adipocytes and *PPAR-α* in the liver and decreasing *SREBP-1c* in the liver. This could be the reason for WEPE’s ability to reduce hepatic fat deposition. These findings exhibited that WEPE could be beneficial for treating HFD-induced steatosis (Tung et al., 2018).

### 5.6 Neuroprotective activity

Flavonoids have garnered attention for their ability to alter neuronal activity and prevent age-related neurodegeneration (Dajas et al., 2003). Flavonoid-rich plant or food extracts may preserve fragile neurons, enhance neuronal function, or stimulate neuronal regeneration to improve cognition in people and animals (Hwang et al., 2012). Their neuroprotective properties have been demonstrated in oxidative stress and Aβ-induced neuronal death models. Ginkgo biloba extracts high in flavonoids have been shown to benefit and modulate the brain, especially in Alzheimer’s disease and age-related dementia (Calderaro et al., 2022). Individual flavonoids, such as the citrus flavanone tangeretin, have been shown to maintain nigro-striatal integrity and functionality after 6-hydroxydopamine lesioning, suggesting that it may protect against Parkinson’s disease pathology (Vauzour et al., 2008). In a recent study conducted by Rajalakshmi et al. (2019), The results of this study on Phyllanthus emblica (Indian gooseberry) offer valuable insights into its potential neuroprotective and antioxidant properties, shedding light on its traditional medicinal uses. This research focused on assessing the radical scavenging capabilities of P. emblica through various assays and its impact on neuroprotection using human neural cell lines (PC12) subjected to glutamate-induced cellular inhibition.

One of the key findings of this investigation was the remarkable antioxidant activity exhibited by P. emblica extract. The DPPH and hydroxyl radical scavenging assays revealed IC50 values of 73.21 μg/mL and 0.426 mg/mL, respectively. This indicates its capacity to effectively neutralize free radicals, which are known to contribute to oxidative stress and various health issues. Moreover, the study observed significant lipid peroxidation activity (IC50: 73.21 μg/mL), further highlighting the potential of P. emblica in preventing oxidative damage to cellular membranes, a crucial factor in maintaining cellular integrity. The neuroprotective effects of P. emblica were also explored, and the results demonstrated its ability to safeguard PC12 cells against glutamate-induced cytotoxicity. This was confirmed through cell viability assays, which showed that P. emblica extract had a protective effect on neural cells. Additionally, monitoring LDH activity, GSH levels, and ROS levels provided further evidence of its neuroprotective properties. According to Li et al. (2011), the extracts of *P. emblica* were found to provide protection to PC12 cells against cell death triggered by H2O2. All samples derived from *P. emblica* exhibited a protective effect on H2O2-induced PC12 cell death, which was both dose-dependent and consistent across all extracts. The results indicate that the hot water and ethanol extracts exhibited superior PC12 cell protection percentages compared to the commercial extracts. According to Li et al. (2022), the neuroprotective benefits of hydroalcoholic extracts derived from *P. emblica* were shown in rat models with kainic acid-induced seizures. These effects may be attributed to the antioxidant and anti-inflammatory properties of the extracts.

### 5.7 Cardioprotective potential

Globally, there is a significant escalation in the prevalence of chronic diseases, including cardiovascular diseases, cancer, diabetes, and obesity. In the year 2001, chronic diseases accounted for nearly 59% of the total recorded deaths worldwide, amounting to 56.5 million fatalities. Furthermore, these disorders were responsible for 46% of the overall burden of disease on a global scale. Cardiovascular diseases (CVD) encompass a range of conditions that affect the heart and blood arteries, such as hypertension (elevated blood pressure) and coronary heart disease (myocardial infarction) (Mayakrishnan et al., 2013). According to Ekta in Pria et al, oral administration of *P. emblica* fruit extract at a dose of 50 and 100 mg/kg BW twice a day for 2 weeks significantly reversed the effects of IRI (ischaemia reperfusion injury), a disease occurs due to oxidative stress. This cardioprotective effect occurs due to the emblicanin A and B contents of *P. emblica* fruit extract (Rajak et al., 2004).

Globally, there has been a concerning surge in the prevalence of chronic diseases, encompassing conditions such as cardiovascular diseases, cancer, diabetes, and obesity. In 2001, chronic diseases accounted for a staggering 59% of total recorded deaths worldwide, resulting in 56.5 million fatalities. Additionally, these ailments imposed a substantial burden, constituting 46% of the overall global disease burden. Among chronic diseases, cardiovascular diseases (CVD) comprise a spectrum of conditions affecting the heart and blood vessels, including hypertension (elevated blood pressure) and coronary heart disease (myocardial infarction) (Mayakrishnan et al., 2013). In a noteworthy study by Ekta in Pria et al., it was demonstrated that the oral administration of *P. emblica* fruit extract at doses of 50 and 100 mg/kg body weight, twice a day for 2 weeks, led to a significant reversal of the effects of ischemia-reperfusion injury (IRI), a condition arising from oxidative stress. This observed cardioprotective effect is attributed to the presence of emblicanin A and B within *P. emblica* fruit extract (Rajak et al., 2004).

### 5.8 Anticancer activity

**FIGURE 6:**
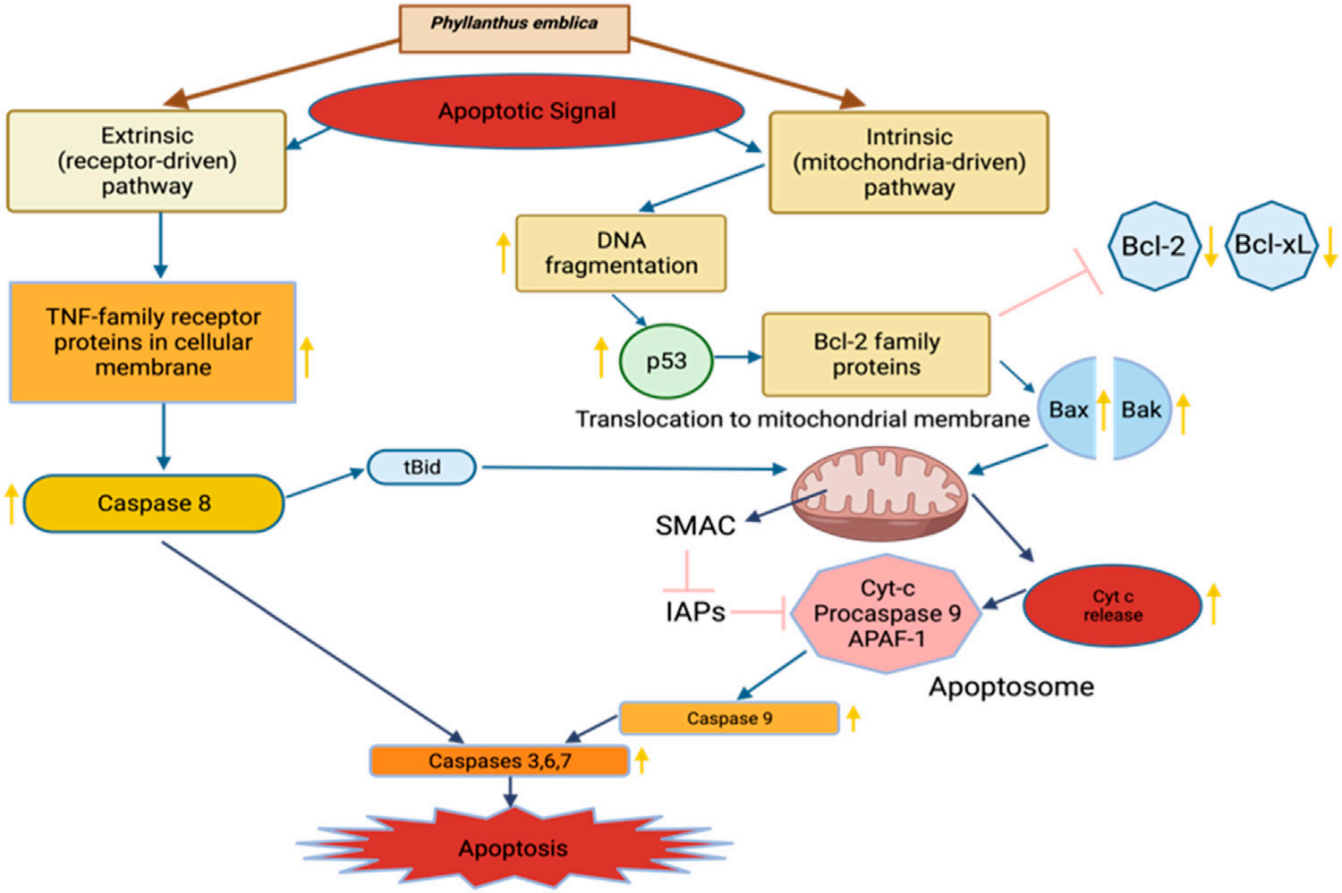
Anticancer of *Phyllanthus emblica*.

Cancer remains a prominent global health challenge, compelling researchers to explore novel substances and treatments aimed at diminishing cancer cell survival, impeding angiogenesis, thwarting proliferation, and restraining metastasis. In the past decade, specific phytochemical compounds and flavonoids have emerged as promising candidates for cancer therapy. In a study conducted by Mahata et al. (2013), the mechanism of action of Phyllanthus emblica fruit extract was meticulously examined. The investigation focused on its impact on activator protein-1 (AP-1) activity and its relevance to cervical cancer cells driven by human papillomavirus (HPV). The findings unveiled a compelling pattern: the Phyllanthus emblica fruit extract exhibited dose- and time-dependent inhibition of DNA binding in both constitutively active AP-1 HPV16-positive (SiHa) and HPV18-positive (HeLa) cervical cancer cells. This AP-1 inhibition, accompanied by the suppression of viral transcription, resulted in the inhibition of cervical cancer cell growth. Moreover, the growth-inhibitory effect of Phyllanthus emblica was primarily attributed to the induction of apoptotic cell death. These findings collectively suggest that Phyllanthus emblica demonstrates its anticancer efficacy by concurrently inhibiting AP-1 and targeting the transcription of viral oncogenes responsible for the development of cervical cancer, thereby indicating its potential utility in the treatment of HPV-induced cervical cancer cells (Mahata et al., 2013; Quranayati et al., 2022). In a separate investigation by Samatiwat et al. (2021), the anti-proliferative activity of the ethanolic extract of Phyllanthus emblica bark was assessed in the context of cholangiocarcinoma. The results highlighted the extract’s cytotoxic potential on the KKU-452 CCA cell line, with an IC50 value of 52.2 μg/mL, accompanied by a significant induction of apoptosis. Furthermore, the ethanolic extract of Phyllanthus emblica bark substantially inhibited cell migration at concentrations of 25 and 50 μg/mL, with reductions of 425% and 32.9%, respectively, compared to untreated cells. These anticancer effects were attributed to the phenolic acid and flavonoid content present in the bark extract of Phyllanthus emblica (Samatiwat et al., 2021). The anticancer potential of Phyllanthus emblica is graphically illustrated in Figure 6.

### 5.9 Antihyperlipidemic and atherogenic activity

In recent years, hyperlipidemia and oxidative stress have emerged as significant health concerns, acknowledged as primary risk factors in the development and progression of atherosclerosis, as well as cardiovascular and cerebrovascular diseases (Li et al., 2011). Consequently, there has been a growing interest in exploring the potential of new natural antioxidants, predominantly of plant origin (Sun et al., 2021). Flavonoids and phenolic compounds derived from plants have gained recognition for their multifaceted pharmacological properties, including antioxidant and anti-hyperlipidemic effects (Gong et al., 2020). The powdered extract of *Phyllanthus emblica* has demonstrated remarkable anti-hyperlipidemic, hypolipidemic, and anti-atherogenic effects, substantiated by statistically significant differences when compared to control groups (Santoshkumar et al., 2013). These effects can be conceivably attributed to the flavonoid content found in Phyllanthus emblica, which exhibits a hypolipidemic effect in atherogenic albino rats. The flavonoids in Phyllanthus emblica function as hypolipidemic agents by inhibiting the activity of HMG-CoA reductase while concurrently increasing the activity of plasma lecithin cholesterol acyl transferase (LCAT) (Sobhani et al., 2017).

### 5.10 Antihypertensive activity

**FIGURE 7:**
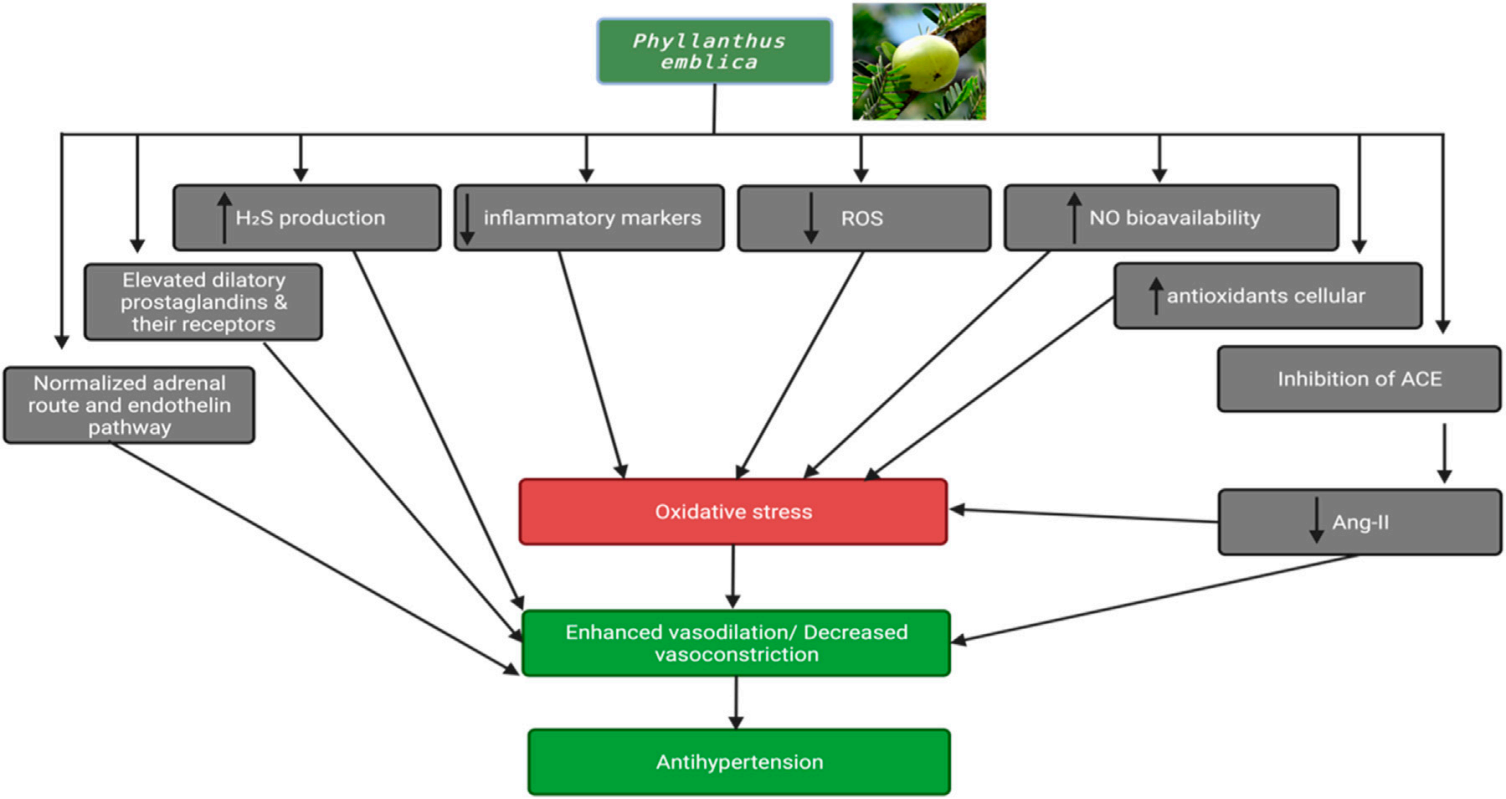
Antihypertension activity of *Phyllanthus emblica*.

In a rigorously conducted study by Ghaffari et al. (2020), a randomized, triple-blind, and placebo-controlled research approach was employed to investigate the antihypertensive effects of *P. emblica*. The study included 92 patients who were randomly assigned to two groups. Each group received either *P. emblica* at a dosage of 500 mg/three times daily after meals or a placebo, in addition to their standard antihypertensive treatments. The findings of this study were quite compelling. After 8 weeks of treatment, the systolic blood pressure exhibited a significant reduction of 15.6% ± 8.23% in the *P. emblica* group, as compared to a 6.3% ± 7.49% decrease in the placebo group. Likewise, diastolic blood pressure showed a notable decrease of 12.3% ± 7.87% in the *P. emblica* group, in contrast to a 3.88% ± 7.98% decrease in the placebo group. Importantly, throughout the study duration, no toxic side effects were observed in any of the patients, underscoring the safety profile of *P. emblica* in this context (Ghaffari et al., 2020). For a visual representation of the mechanism, please refer to Figure 7.

### 5.11 Antimicrobial activity

Jahan and Akter (2015) conducted research to evaluate the antimicrobial activity of *Phyllanthus emblica*. This research was conducted utilizing disc diffusion method. The methanolic extract of *P. emblica* was assayed at 500 µg/disc concentration with standard kanamycin disc against gram positive, gram negative and multi drug resistant strains. The results demonstrated that the ethanolic extract of PE exhibited significant antimicrobial effect againts some microorganisms especially, *Bacillus subtilis* (25 mm), *Staphylococcus aureus* (20 mm) and *Shigella dysenteriae* (17 mm). Both aqueous and ethanolic extract of *P. emblica* was proven effective against *Pseudomonas aeruginosa* in disc diffusion method (Farhana et al., 2022). The methanolic extract of *P. emblica* at a dose rate of 50 mg/mL and 25 mg/mL, respectively, had a complete bactericidal effect on AMR (antimicrobial-resistant) *S. Typhi* and S. Enteritidis (Nair et al., 2020).

### 5.12 Anti-diarrheal activity

According to Afrin et al. (2016), *P. emblica* extract exhibited a significant anti-diarrheal effect. These authors conducted research to evaluate the anti-diarrheal activity towards diarrheal (castor oil induced) mice at a dose of 2 mL/mouse. The extracts of *P. emblica* at a dose of 500 mg/kg BW were given 1 h before the mice were induced with castor oil orally. The results showed that *P. emblica* significantly inhibited the defecation mean number compared to control group administrated with standard anti-diarrheal drug. The methanolic extract of *P. emblica* at a dose of 25% exhibited 42.86% inhibition while at a dose of 500 mg/kg BW, it showed 64.29% inhibition. The anti-diarrheal standard drug (loperamide) at a dose of 5 mg/kg exhibited 71.43% inhibition. As *P. emblica* succesfully inhibited the castor oil induced diarrhea, it might have exhibited its antidiarrheal effect by inhibiting the biosynthesis of prostaglandin through antisecretory mechanism (Afrin et al., 2016).

### 5.13 Anti-aging activity

In a study conducted by Wu et al. (2022), it was observed that the polyphenols found in the fruit of *P. emblica* demonstrated a notable protective effect against the aging process in the *Caenorhabditis elegans* model. The anti-aging properties were evidenced through the augmentation of thermal resistance, as well as the significant reduction in the activity levels of acetylcholinesterase (AChE) by 34.71% and butyrylcholinesterase (BuChE) by 45.38%. These findings were accompanied by a significant increase in the activity of antioxidant enzymes, specifically superoxide dismutase (SOD) by 30.74% and catalase (CAT) by 8.42%. Additionally, there was a notable decrease in the amount of malondialdehyde (MDA) by 36.25%. The presence of rich flavonols and phenolic acids, including quercetin, myricetin, ellagic, gallic, and chlorogenic acids, together with their glycosides, may potentially be associated with the interrelation of these qualities (Wu et al., 2022).

### 5.14 Laxative

Mehmood et al. (2013) reported that the crude extract derived from *P. emblica* exhibited prokinetic and laxative properties in mice. The extract facilitated the movement of charcoal meal through the small intestine and resulted in an increase in the production of wet feces. These effects were comparable to the effects observed with carbachol, a commonly used cholinergic agonist known for its ability to accelerate intestinal contents. The extract’s gut stimulatory activities were observed to be slightly influenced by atropine, a blocker of muscarinic receptors. The mice exhibited wet feces at a rate of 35.7% and 44.1% when administered with a crude extract of *P. emblica* at doses of 100 and 300 mg/kg, respectively. The experimental group receiving the positive control, CCh (1 mg/kg), exhibited a moist feces percentage of 48.2%, whereas the group treated with saline solution displayed a wet feces percentage of just 10.5%. The laxative effect was determined by calculating the percentage increase in moist feces compared to the total fecal production, as described by Mehmood et al. (2013). The pharmacological activity of *P. emblica* can be seen in Table 2.

**TABLE 2:** Pharmacological effects and mechanisms of action of extract from *Phyllanthus emblica*.

No.	Dose	Pharmacology activity	Mechanism	References
1	50 and 100 μg/mL	Antioxidant	- enhancing reducing power and total antioxidant capacity, scavenging hydroxil radical and superoxide anion	Uddin et al. (2016); Li et al. (2020)
			- Reduced peroxidation level	
			- Induced defense system (GSH, SOD, CAT, GPx, GSH reductase, and GSH S-transferase)	
2	50 and 100 μg/mL	Antiinflammatory	- Inhibited Nitric Oxide (NO) and COX-2 (dose-dependent)	Li et al. (2022)
3	100; 200; 500 mg/kg BW	Immunomodulatory	- Induced the activity of GSH, CAT, and SOD in the thymus of mice	Ghosh et al. (2013); Saini et al. (2022)
			- increased the hemagglutination antibody titer, leukocytes count, the percentage of lymphocytes distribution, and delayed hypersensitivity in mice	
4	500 mg/kg BW	Hepatoprotective	- Gallic acid content improves high fat diet induced dyslipidemia, hepatosteatosis, and oxidative stress	Huang et al. (2017)
			- increasing *adiponectin* in adipocytes and *PPAR-α* in the liver and decreasing *SREBP-1c* in the liver	
5	Dose dependent	Neuroprotective	- Conferred PC12 cells protection against H_2_O_2_-induced cell death	Li et al. (2022)
6	50 and 100 mg/kg BW	Cardioprotective	- The emblicanin A and B contents reversed the effects of IRI (ischaemia reperfusion injury)	Priya and Islam (2019)
7	25 and 50 μg/mL	Anticancer	- Phenolic acid and flavonoid content inhibited cell migration	Samatiwat et al. (2021)
8	2 mL/kg/day	Antihyperlipidemic	- Activated PPARα and carnitine palmitoyl transferase (involved in lipid oxidation)	Variya et al. (2016)
			- Reduced the serum triglycerides	
9	500 mg/TDS	Antihypertensive	- Reduced systolic and diastolic blood pressure	Ghaffari et al. (2020)
10	50 and 25 mg/mL	Antimicrobial	- Methanolic extract showed potent antimicrobial activity to antimicrobial-resistant (AMR) *Salmonella Typhi* and *Salmonella Enteritidis*; *Bacillus subtilis*, *Staphylococcus aureus* and *Shigella dysenteriae*; *Pseudomonas aeruginosa*	Nair et al. (2020); Jahan and Akter (2015); Farhana et al. (2022)
11	250 and 500 mg/kg BW	Anti-diarrheal	- Inhibited the biosynthesis of prostaglandin through antisecretory mechanism	Afrin et al. (2016)
12	1.2 mg/mL	Anti-aging	- Reduced the activity of AchE, BuChE; enhanced antioxidant enzymes activity of SOD and CAT; decrease MDA level	Wu et al. (2022b)
13	100 and 300 mg/kg BW	Laxative	- The crude extract increased the production of wet feces at a mechanism similar to the effect of standard drug carbachol	Mehmood et al. (2013)

## 6 Safety and toxicity

Plants especially medicinal plants have been used for treatments in humans. Since the prehistoric era, raw parts or extract of *Phyllanthus emblica* have been used to treat various diseases and *in vitro* and *in vivo* studies have proven its effectiveness to inhibit various pathogenesis. Chronic toxicity studies by inducting *P. emblica* oral doses of 300, 600 and 1,200 mg/kg for 270 days resulted in no evident changes in treated animals pathologically. Previous studies reported the absence of toxicity of *P. emblica* fruit extract at doses of 200, 400, 300, and 500 mg/kg (Golechha et al., 2014; Middha et al., 2015; Anto et al., 2022). Furthermore, Uddin et al. (2016) reported that the ethanolic extract of Phyllanthus emblica is safe with a dose up to 2000 mg/kg b.w. in rats. The hematological examination, behavioral observation, and biochemical test of *P. emblica* demonstrated the absence of toxic effect. Thiennimitr et al. (2018) reported that consuming *Lactobacillus* sp. mediated fermented *P. emblica* fruit juice up to 9 mL/kg/day for 60 days to both male and female rats is safe and no rat exhibited any remarkable changes in the body weights, internal organs, hematology, and biochemical parameters.

## 7 Future perspective and challaenges

Plants have been used as remedial agents throughout history. In the last two centuries, scientists have found a way to utilize secondary metabolites contents in medicinal plants for drug manufacturing. *Phyllanthus emblica*, is a widely used herb in the Indian medicinal system. Numerous researches have proven therapeutic properties using different extracts and herbal preparations of *P. emblica*. A variety of well-established beneficial health effects and pharmacological activities was ascribed to *P. emblica*, namely, antioxidant, anti-cancer, hepatoprotective, neuroprotective, immunomodulatory, anti-inflammatory, anti-diabetic, and anti-hyperlipidemic effects. *Phyllanthus emblica* is traditionally used to address numerous disorders along with food ingredients. In spite of the fact that various modern research techniques have been established to validate the medicinal uses of *P. emblica* traditionally, some aspects including its contents and its applications need to be further investigated scientifically. For example, only a few studies reported *P. emblica* antimalarial, antiviral, anti-venom, and insecticidal properties. Some of its properties were also reported with other parts of *P. emblica*. Accordingly, it is imperative that the agents, molecules or parts mediating its therapeutic activities be identified. Additionally, more extensive research, such large-scale evidence-based trials, must be done to examine the medical benefits of *P. emblica*.

## 8 Conclusion

In conclusion, our comprehensive review of *P. emblica*, commonly known as Indian gooseberry or Amla, has shed light on its remarkable phytochemical composition and extensive pharmacological properties. This indigenous fruit, deeply rooted in traditional medicine, has proven to be a valuable source of bioactive compounds that hold immense potential for various therapeutic applications. The phytochemical analysis of Phyllanthus emblica revealed a rich array of secondary metabolites, including flavonoids, tannins, polyphenols, and ascorbic acid. These compounds collectively contribute to the antioxidant, anti-inflammatory, and antimicrobial activities of the plant, making it a promising candidate for the development of natural remedies and pharmaceutical agents. Regarding its pharmacological properties, Phyllanthus emblica has exhibited a wide spectrum of effects, ranging from its hepatoprotective and immunomodulatory actions to its antidiabetic, anticancer, and cardioprotective potentials. The extensive research conducted in this area has highlighted the versatility of this botanical treasure, providing avenues for future investigations and applications in the realm of medicine and healthcare. However, it is essential to acknowledge the limitations of our review. Firstly, while we have compiled a comprehensive overview of the available literature, the field of *P. emblica* research is continuously evolving, and new discoveries may have emerged since the completion of this review. Additionally, the majority of studies have been conducted *in vitro* or in animal models, necessitating further clinical trials to establish the efficacy and safety of Phyllanthus emblica-based interventions in humans. Moreover, variations in the phytochemical composition of *P. emblica* due to geographical and environmental factors pose a challenge in standardizing its therapeutic applications. *Phyllanthus emblica* holds great promise as a source of bioactive compounds with diverse pharmacological properties. While this review serves as a valuable resource for understanding its potential benefits, further research and clinical investigations are warranted to fully harness the therapeutic potential of this remarkable botanical species. Despite the limitations, the compelling evidence presented in this review underscores the significance of Phyllanthus emblica in the realm of natural medicine and inspires continued exploration in the pursuit of improved healthcare solutions.
